# Trends in metastatic thyroid cancer survival from 1992 to 2022: a SEER database analysis

**DOI:** 10.1530/EO-25-0086

**Published:** 2026-05-20

**Authors:** Ashwin Govindan, Philippos Apolinario Costa, Richard Feinn, Hari Deshpande

**Affiliations:** ^1^ Frank H Netter MD School of Medicine, North Haven, Connecticut, USA; ^2^ Yale School of Medicine, New Haven, Connecticut, USA

**Keywords:** carcinoma, intracellular signaling, metastasis, oncology, thyroid

## Abstract

The majority of thyroid cancers present as localized disease that is treated with surgery or radioactive iodine. Despite the effectiveness of radioactive iodine, 5–10% of thyroid cancers are radioiodine-resistant and are termed radioiodine-refractory (RAIR) thyroid cancers. RAIR tumors that spread distantly exhibit high mortality. Phase III trials, in the early 2010s, for tyrosine kinase inhibitors (TKIs) such as lenvatinib, sorafenib, and cabozantinib have shown promise in extending progression-free survival (PFS) in patients with RAIR, but it remains unclear whether these advances have improved disease-specific survival (DSS). We conducted a survival analysis of metastatic thyroid cancers using the Surveillance, Epidemiology, and End Results (SEER) national database to assess changes in DSS from 1992 to 2022. Patients were divided into three cohorts based on the year of diagnosis: 1992–2001, 2002–2011, and 2012–2022. Kaplan–Meier and Cox regression analyses were used to assess survival trends and effects of demographic and treatment variables. Our results suggest a modest but significant improvement in DSS for those diagnosed with distant metastasis of anaplastic, follicular, medullary, and oncocytic thyroid carcinoma in the most recent decade (2012–2022) compared to earlier periods. Limited information on detailed treatment data, imposed by the use of SEER, ought to be considered. Future studies with more detailed treatment data would be beneficial for confirming our findings and better characterizing RAIR thyroid cancer survival.

## Introduction

The estimated incidence of thyroid cancer in the United States is 44,020 cases per year (1). Papillary (84%), follicular (4%), and oncocytic (2%) thyroid carcinomas are referred to as differentiated thyroid cancers (2). Differentiated thyroid cancers account for 90% of all newly diagnosed thyroid malignancies. The 5-year overall survival for all thyroid cancers is 98.5% (2). The majority of thyroid cancers present as localized disease, with surgery and radioactive iodine remaining the cornerstones of therapy. Despite the effectiveness of radioactive iodine in differentiated thyroid cancers, 5–10% of thyroid cancers are radioiodine-refractory (RAIR) thyroid cancers and further classified into localized and distant metastatic disease based upon the extent of tumor invasion (3). Other types of thyroid tumors (e.g. anaplastic and medullary thyroid cancers) make up a significant portion of thyroid cancer-related mortality despite only comprising 1–2% and 3–5% of thyroid tumors, respectively (4). The mean survival for anaplastic tumors is 6 months, and the 10-year survival rate for distantly metastasized medullary thyroid tumors remains at 40% (5, 6). Many of these tumors are refractory and respond poorly to traditional chemotherapies, necessitating the development of targeted therapies (7).

The introduction of tyrosine kinase inhibitors (TKI) as a form of first- or second-line treatment for refractory thyroid cancers has been a significant advancement for aggressive forms of thyroid cancer. These treatments target alterations in vascular endothelial growth factor (VEGF) and v-Raf murine sarcoma viral oncogene homolog B1 (BRAF) (8). Phase III trials for TKIs such as lenvatinib, sorafenib, and cabozantinib have shown promise in extending progression-free survival (PFS) in patients with RAIR tumors. A trial of lenvatinib, published in 2015, found a median PFS of 18.3 months in the lenvatinib group compared to 3.6 months in the control group (8). Similarly, a phase III trial of sorafenib found a median PFS extension of 5 months compared to placebo (9). In 2021, Brose *et al.* reported statistically significant improvement in PFS in patients with RAIR tumors with cabozantinib compared to placebo (9). The US Food and Drug Administration (FDA) approved the use of sorafenib, lenvatinib, and cabozantinib in 2012, 2015, and 2021, respectively (8, 9, 10). The combination of dabrafenib and trametinib yielded an overall response rate of 69% in *BRAF*-mutant anaplastic thyroid cancer (11). Despite the improved outcomes in median progression-free survival (PFS) demonstrated in these large-scale clinical studies, it is unclear whether there have been improvements in disease-specific survival (DSS) across the various thyroid cancer subtypes in population-based studies.

This study investigated DSS in patients with metastatic thyroid carcinomas using the Surveillance, Epidemiology, and End Results (SEER) database to determine whether DSS has changed over the past three decades.

## Methods

We compared DSS in patients with all types of metastatic thyroid cancer (using those cancers tagged as ‘distant’ in SEER) from 1992 to 2022 to explore if survival has changed in this time period. We then compared DSS between different groups (Groups 1, 2, and 3) corresponding to the following time periods: 1992–2001, 2002–2011, and 2012–2022. The last period, 2012–2022, was chosen to encompass all years in which TKIs were used in thyroid cancer treatment.

The SEER 12 database collects data from 12 geographic areas (San Francisco-Oakland, Connecticut, Hawaii, Iowa, New Mexico, Seattle, Utah, Atlanta, San Jose-Monterey, Los Angeles, Alaska Natives, and Rural Georgia) from 1992 to 2022. The case listing function (i.e. individual, anonymized patient records from the SEER database) was used in the SEER 12 database to generate all applicable cases for analysis. The inclusion criteria for generating case data included thyroid as the primary site of cancer and the presence of ‘distant’ disease. Due to incomplete TNM staging data, SEER summary staging data were used. SEER summary staging uses all available clinical data for a given patient to determine whether a patient’s disease status fits into localized, regional, distant, or unknown. ‘Distant’ disease status was defined in SEER as ‘metastatic’ or ‘advanced’ disease, and only cases with this status were included in the analysis. ‘Distant’ disease status had three definitions within the SEER 12 database: ‘SEER Historic Stage A’ (1973–2015), ‘Summary Stage’ (1998–2017), and ‘Combined Summary Stage’ (2004+); the database was then queried for thyroid as the primary site of disease and ‘distant’ disease status per any of the aforementioned SEER 12 definitions. For the sake of consistency, distant disease status is referred to as ‘metastatic’ in this manuscript.

### Statistical analyses

ICD-10 codes were used to sort all queried SEER data into five categories by thyroid cancer subtype. These categories and the associated codes are as follows: papillary (ICD-10: 8,050, 8,052, 8,260, 8,340–8,344, 8,350), follicular (ICD-10: 8,330–8,334), oncocytic (ICD-10: 8,290, 8,335), medullary (ICD-10: 8,345, 8,510), and anaplastic (ICD-10: 8,020–8,023, 8,030–8,035). All remaining data that did not otherwise fall into any of the aforementioned categories was tagged ‘not otherwise classified (NOC)’. Survival was defined as DSS, equal to the time from diagnosis to death from disease. Patients who had died from some other causes or were alive at the time of the last recording were censored.

Due to vastly different tumor characteristics, disease prognosis, and survival outcomes, thyroid tumors were separated into differentiated and other thyroid tumors. Differentiated thyroid tumors were selected for and analyzed as part of the primary analysis and included papillary, follicular, and oncocytic tumors. Medullary, anaplastic, and not otherwise classified (NOC) tumors were analyzed as part of a secondary analysis and comprised other thyroid tumors. Demographic and survival analyses were stratified by differentiated vs other classifications; survival calculations were further stratified by histologic subtype. A further analysis was conducted for treatment effect on MTC. Median DSS was not reached in several subgroups due to low event rates (i.e. high survival or low sample size). As a result, survival analyses were primarily assessed using Cox proportional hazards ratios and log-rank tests, stratified by histology, with Kaplan–Meier curves presented for visualization. All statistical analyses were conducted using SPSS V29.0.

## Results

### Demographics

A total of 4,781 patients from 1992 to 2022 with metastatic, differentiated thyroid cancers were identified, with 61.4% being female and 38.6% male. Most patients were White (73.3%), followed by Asian or Pacific Islander at 19%; Black individuals comprised 5.6%. Patients of unknown race and American Indian/Alaska Native race comprised smaller percentages at 0.5 and 1.6%, respectively; 64.5% patients were 50 years of age or older. There were statistically significant differences between the three time periods for the demographic variables: age, race, and sex (Table 1). Notably, statistically significant differences were observed between time periods for treatment with chemotherapy and radiation (*P* < 0.001). Chemotherapy was coded as either yes or no/unknown, with 5.7% having received chemotherapy (defined broadly and including cytotoxic chemotherapy, immunotherapy, and targeted therapies).

**Table 1:** Demographic summary for individual groups, percentages by time period (PTC, FTC, and OTC).

	Group 1 (1992–1999)	Group 2 (2000–2009)	Group 3 (2010–2021)	Total	Differences between groups
Sex					*P < 0.001*
Female	714 (63.1%)	1,222 (64.2%)	998 (57.2%)	2,934 (61.4%)	
Male	417 (36.9%)	682 (35.8%)	748 (42.8%)	1847 (38.6%)	
Age (years)					*P < 0.001*
05–19	48 (4.2%)	52 (2.7%)	39 (2.2%)	139 (2.9%)	
20–34	186 (16.4%)	261 (13.7%)	181 (10.4%)	628 (13.1%)	
35–49	238 (21.0%)	403 (21.2%)	289 (16.6%)	930 (19.2%)	
50–64	256 (22.6%)	483 (25.3%)	510 (29.2%)	1,249 (26.1%)	
65–79	312 (27.6%)	516 (27.1%)	525 (30.1%)	1,353 (28.3%)	
80+	91 (8.0%)	189 (9.9%)	202 (11.6%)	482 (10.1%)	
Race					*P = 0.022*
American Indian/Alaska Native	17 (1.5%)	30 (1.6%)	29 (1.7%)	76 (1.5%)	
Asian or Pacific Islander	183 (16.2%)	363 (19.1%)	363 (20.8%)	909 (19.0%)	
Black	57 (5.1%)	101 (5.3%)	109 (6.2%)	267 (5.6%)	
Unknown	3 (0.3%)	9 (0.4%)	14 (0.8%)	26 (0.5%)	
White	871 (77.0%)	1,401 (73.6%)	1,231 (70.5%)	3,503 (73.3%)	

A total of 1966 patients from 1992 to 2022 (Table 2) with metastatic, other thyroid cancers were identified, with 55.8% being female and 44.2% male. Most patients were White (75.3%), followed by Asian or Pacific Islander at 15.3%; Black individuals comprised 8.2%. Patients of unknown race and American Indian/Alaska Native race comprised smaller percentages at 0.25 and 0.96%, respectively; 88.9% patients were 50 years of age or older. There were statistically significant differences between the three time periods for the demographic variables: age and race. Notably, neither treatment with chemotherapy nor radiation was significantly different by time period for other thyroid cancers.

**Table 2:** Demographic summary for individual groups, percentages by time period (MTC, ATC, and others).

	Group 1 (1992–1999)	Group 2 (2000–2009)	Group 3 (2010–2021)	Total	Differences between groups
Sex					*P = 0.155*
Female	234	368	495	1,097	
Male	155	304	410	869	
Age (years)					*P = 0.019*
05–19	4	4	0	8	
20–34	13	18	14	45	
35–49	39	66	60	165	
50–64	96	169	250	515	
65–79	149	261	379	789	
80+	88	154	202	444	
Race					*P < 0.001*
American Indian/Alaska Native	3	8	8	19	
Asian or Pacific Islander	43	96	161	300	
Black	25	48	89	162	
Unknown	0	0	5	5	
White	318	520	642	1,480	

### Survival analysis

A Cox regression model (Table 3) was used to assess the effect of covariates on survival. The model included age, sex, race, time period of diagnosis (Group 1: 1992–2001, Group 2: 2002–2011, and Group 3: 2012–2022; Group 1 used as reference), and was stratified by individual histologic subtype (with the primary analysis pertaining to all histologic subtypes within differentiated thyroid tumors). Across all differentiated subtypes, younger age was strongly associated with increased DSS (*P* < 0.001), with age groups 0–19, 20–34, and 35–49 having hazard ratios (HRs) of 0.028, 0.018, and 0.112, respectively, compared to the >80 age group. Female sex (HR = 0.74, *P* < 0.001) and later diagnostic eras (Groups 2 and 3: HR = 0.80, *P* < 0.001) were associated with decreased mortality. Treatment with chemotherapy was associated with decreased mortality (HR = 0.449, *P* < 0.001), while treatment with radiation was not significantly associated with mortality.

**Table 3:** Histological subtypes and percentages by groups.

	Group 1 (1992–1999)	Group 2 (2000–2009)	Group 3 (2010–2021)	Total	Differences between groups
Histologic subtype					*P < 0.001*
Anaplastic	164	280	435	879	
Follicular	155	178	195	528	
Medullary	75	113	145	333	
Oncocytic	31	66	47	144	
Papillary	937	1,656	1,498	4,091	
Others	148	279	324	751	

**Figure 1:**
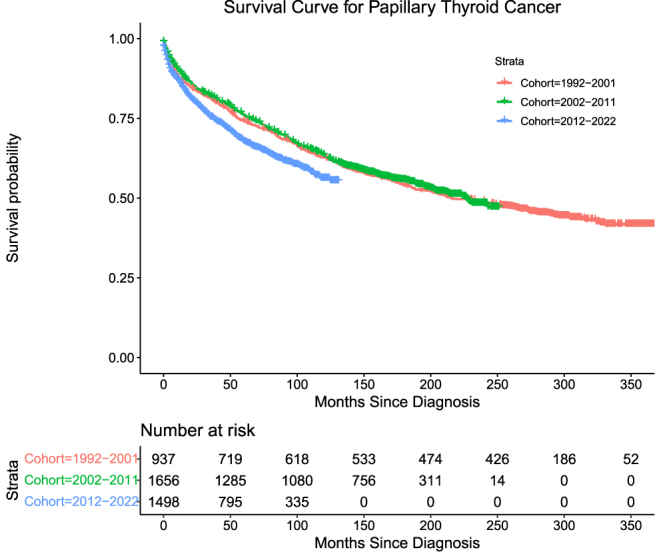
Survival curve for papillary thyroid cancer.

Papillary thyroid cancers (PTC) accounted for 85.9% (*n* = 4,091) of the cohort. The number of deaths attributable to distant PTC in this cohort was 1,089 (26.6%) overall. Mortality for Groups 1, 2, and 3, respectively, is as follows: 296 (68.4%), 435 (26.3%), and 358 (23.9%) with log-rank (Mantel–Cox) = 19.497, *P* < 0.001. PTC exhibited high DSS across all three groups, with Group 2 having the highest DSS, followed by Group 3, and Group 1 demonstrating the lowest (Table 4, Fig. 1).

**Table 4:** Cox regression for differentiated tumors (PTC, FTC, and OTC).

	*B*	SE	df	*P*	HR	95% CI for HR	
						Lower	Upper
Age (80+)*			5	<0.001			
Age (0–19)	−3.564	0.454	1	<0.001	0.028	0.012	0.069
Age (20–34)	−4.045	0.277	1	<0.001	0.018	0.010	0.030
Age (35–49)	−2.193	0.112	1	<0.001	0.112	0.090	0.139
Age (50–64)	−1.235	0.082	1	<0.001	0.291	0.247	0.342
Age (65–79)	−0.649	0.074	1	<0.001	0.522	0.452	0.604
Sex (male)*			1	<0.001			
Sex (female)	−0.305	0.054	1	<0.001	0.737	0.664	0.819
Race (White)*			4	0.358			
Race (AIAN)	0.097	0.233	1	0.678	1.102	0.698	1.739
Race (AAPI)	−0.092	0.067	1	0.172	0.912	0.799	1.041
Race (Black)	0.086	0.117	1	0.461	1.090	0.867	1.369
Race (unknown)	−1.195	1.001	1	0.233	0.303	0.043	2.154
Radiation (none)*			7	<0.001			
Radiation (beam)	−0.105	0.210	1	0.616	0.900	0.597	1.358
Radiation (combination)	−0.760	0.230	1	<0.001	0.468	0.298	0.734
Radiation (none/unknown)	−0.613	0.207	1	0.003	0.542	0.361	0.813
Radiation (NOS)	−0.168	0.391	1	0.668	0.845	0.393	1.820
Radiation (implants)	−1.411	0.336	1	<0.001	0.244	0.126	0.471
Radiation (radioisotopes)	−1.351	0.207	1	<0.001	0.259	0.173	0.388
Radiation (recommended)	−0.730	0.274	1	0.008	0.482	0.282	0.825
Chemotherapy (no)*			1	<0.001			
Chemotherapy (yes)	−0.801	0.092	1	<0.001	0.449	0.375	0.537
Time period (1992–2001)*			1	<0.001			
Time period (2002–2011)	−0.227	0.066	1	<0.001	0.797	0.701	0.907
Time period (2012–2022)	−0.228	0.072	1	<0.001	0.796	0.692	0.916

* Denotes reference group.

**Figure 2:**
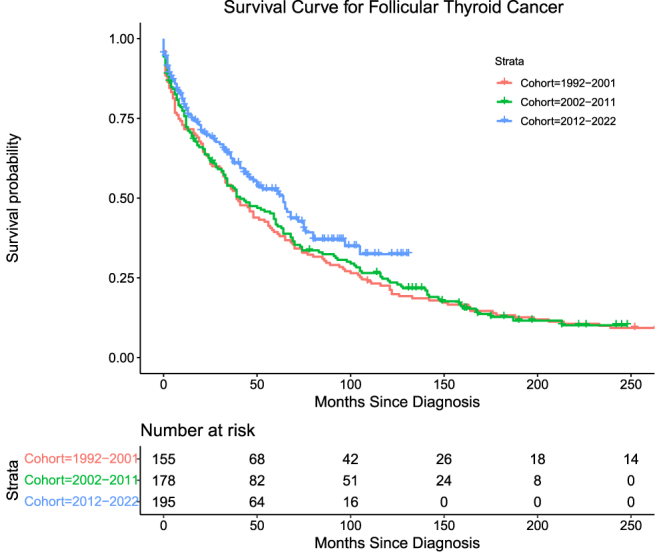
Survival curve for follicular thyroid cancer.

Follicular thyroid cancers (FTC) accounted for 11.1% (*n* = 528) of the cohort. The number of deaths attributable to FTC in this cohort was 279 (52.8%) overall. Mortality for Groups 1, 2, and 3, respectively, is as follows: 107 (69.3%), 106 (59.6%) and 66 (33.8%), with log-rank (Mantel–Cox) = 5.857, *P* = 0.053. FTC exhibited moderate DSS across all three groups, with Group 3 exhibiting the highest DSS, followed by Group 2 and then Group 1 (Table 4, Fig. 2).

**Figure 3:**
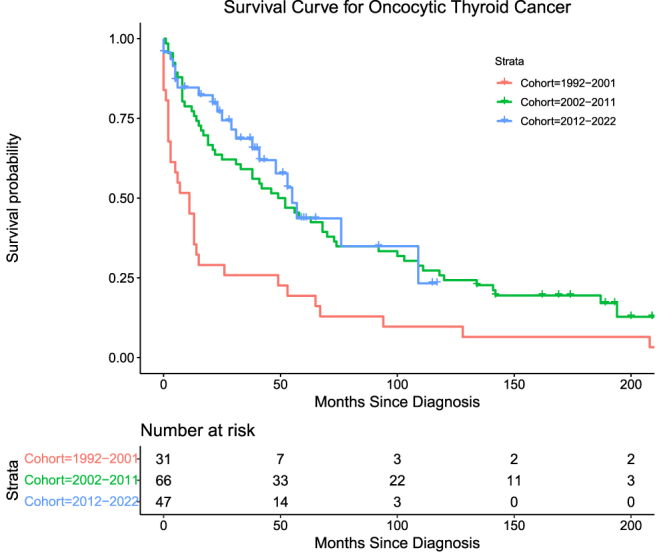
Survival curve for oncocytic thyroid cancer.

Oxyphilic thyroid cancers (OTC) accounted for 3.0% (*n* = 144) of the cohort. The number of deaths attributable to OTC in this cohort was 76 (52.8%) overall. Mortality for Groups 1, 2, and 3, respectively, is as follows: 23 (74.2%), 39 (59.1%), and 14 (29.8%) with log-rank (Mantel–Cox) = 16.195, *P* < 0.001. OTC exhibited moderate DSS across all three groups, with Groups 2 and 3 exhibiting significantly higher DSS than Group 1 (Table 4, Fig. 3).

The same Cox regression model (Table 5) with identical covariates as the differentiated cancers was used to assess the effect of covariates on survival, again stratified by each histologic subtype for other tumors. Across other subtypes, younger age was strongly associated with survival (*P* < 0.001), with age groups 5–19, 20–34, 35–49, and 50–64 having hazard ratios (HRs) equal to 0.130, 0.519, 0.417, and 0.617, respectively, compared to the >80 group. Female sex (HR = 0.897, *P* = 0.05) and later diagnostic eras (Group 2: HR = 0.85, *P* = 0.02; Group 3: HR = 0.79, *P* < 0.001) were associated with decreased mortality. Treatment with chemotherapy was associated with increased mortality (HR = 1.69, *P* < 0.001) while treatment with radiation was not significantly associated with mortality.

**Table 5:** Cox regression for other tumors (MTC, ATC, and NOC).

	*B*	SE	df	*P*	HR	95% CI for HR	
						Lower	Upper
Age (80+)*			5	<0.001			
Age (0–19)	−2.037	0.713	1	0.004	0.130	0.032	0.527
Age (20–34)	−0.655	0.213	1	0.002	0.519	0.342	0.788
Age (35–49)	−0.875	0.129	1	<0.001	0.417	0.324	0.536
Age (50–64)	−0.483	0.081	1	<0.001	0.617	0.526	0.723
Age (65–79)	−0.265	0.070	1	<0.001	0.767	0.669	0.880
Sex (male)*			1	<0.001			
Sex (female)	−0.109	0.055	1	0.049	0.897	0.805	0.999
Race (White)*			4	<0.001			
Race (AIAN)	−0.023	0.293	1	0.936	0.977	0.550	1.734
Race (AAPI)	0.048	0.076	1	0.531	1.049	0.903	1.218
Race (Black)	0.021	0.104	1	0.843	1.021	0.833	1.251
Race (unknown)	−0.790	0.710	1	0.266	0.454	0.113	1.825
Radiation (none)*			7	<0.001			
Radiation (beam)	−0.047	0.202	1	0.817	0.954	0.642	1.419
Radiation (combination)	−0.310	0.327	1	0.343	0.733	0.386	1.392
Radiation (none/unknown)	0.094	0.202	1	0.641	1.099	0.739	1.634
Radiation (NOS)	−0.045	0.406	1	0.911	0.956	0.431	2.117
Radiation (implants)	−0.290	0.613	1	0.636	0.748	0.225	2.489
Radiation (radioisotopes)	−0.871	0.264	1	<0.001	0.419	0.250	0.702
Radiation (recommended)	−0.378	0.350	1	0.280	0.685	0.345	1.361
Chemotherapy (no)*			1	<0.001			
Chemotherapy (yes)	0.523	0.063	1	<0.001	1.688	1.491	1.911
Time period (1992–2001)*			2	0.004			
Time period (2002–2011)	−0.168	0.073	1	0.022	0.845	0.732	0.976
Time period (2012–2022)	−0.239	0.073	1	<0.001	0.787	0.683	0.907

* Denotes reference group.

**Figure 4:**
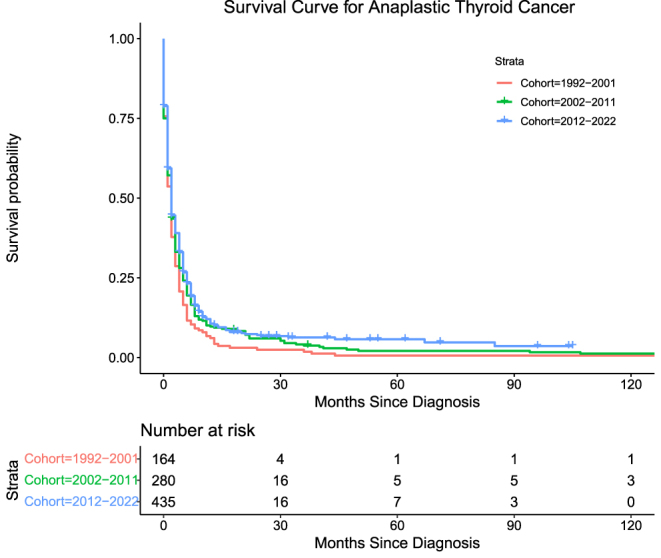
Survival curve for anaplastic thyroid cancer.

Anaplastic thyroid cancers (ATC) accounted for 44.8% (*n* = 879) of the cohort. The number of deaths attributable to ATC in this cohort was 777 (88.4%) overall. Mortality for Groups 1, 2, and 3, respectively, is as follows: 152 (93.3%), 256 (91.4%), and 368 (84.6%) with log-rank (Mantel–Cox) = 8.181, *P* = 0.017. ATC exhibited the lowest DSS of any thyroid cancer subtype, with Group 3 exhibiting moderately higher DSS than Group 2, and Group 1 demonstrating the lowest (Table 5, Fig. 4).

**Figure 5:**
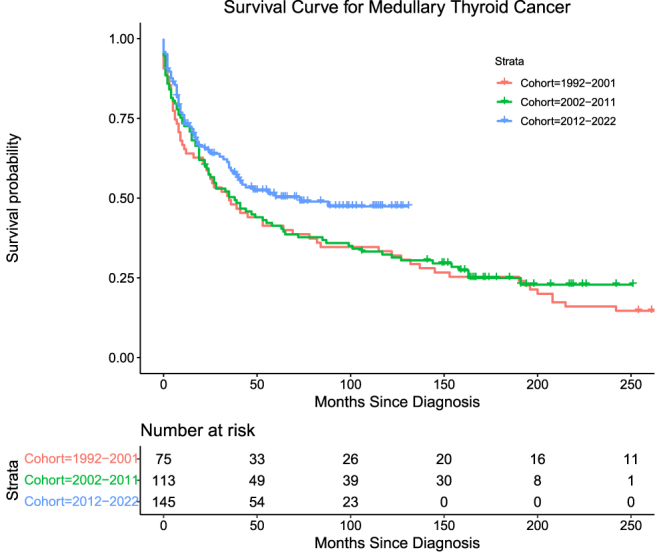
Survival curve for medullary thyroid cancer.

Medullary thyroid cancers (MTC) accounted for 17.0% (*n* = 333) of the cohort. The number of deaths attributable to MTC in this cohort was 172 (51.6%) overall. Mortality for Groups 1, 2, and 3, respectively, is as follows: 52 (69.3%), 66 (58.4%), and 54 (37.2%) with log-rank (Mantel–Cox) = 5.493, *P* = 0.064. MTC exhibited low DSS across all three groups, with the highest DSS observed in Group 3, followed by Group 2, and Group 1 demonstrating the lowest (Tables 5 and 6, Fig. 5). Patients in Group 3 had significantly improved DSS compared to Group 1 patients (HR = 0.492, *P* < 0.001), and patients with MTC demonstrated an elevated HR associated with chemotherapy (HR = 2.121, *P* < 0.001) with no significant effects associated with radiation therapy (Tables 5 and 6, Fig. 5).

**Table 6:** Cox regression for MTC for treatment type and time period.

	*B*	SE	df	*P*	HR	95% CI for HR	
						Lower	Upper
Radiation (beam)*			5	0.145			
Radiation (combination)	0.514	0.741	1	0.488	1.672	0.391	7.149
Radiation (none/unknown)	−0.265	0.165	1	0.108	0.767	0.555	1.059
Radiation (radioisotopes)	−0.615	0.524	1	0.241	0.541	0.194	1.512
Radiation (recommended)	1.288	1.032	1	0.212	3.626	0.480	27.399
Radiation (refused)	1.226	1.038	1	0.238	3.408	0.445	26.083
Chemotherapy (no)*			1	<0.001			
Chemotherapy (yes)	0.752	0.181	1	<0.001	2.121	1.487	3.025
Time period (1992–2001)*			2	0.002			
Time period (2002–2011)	−0.185	0.193	1	0.336	0.831	0.569	1.212
Time period (2012–2022)	−0.710	0.211	1	<0.001	0.492	0.325	0.744

* Denotes reference group.

**Figure 6:**
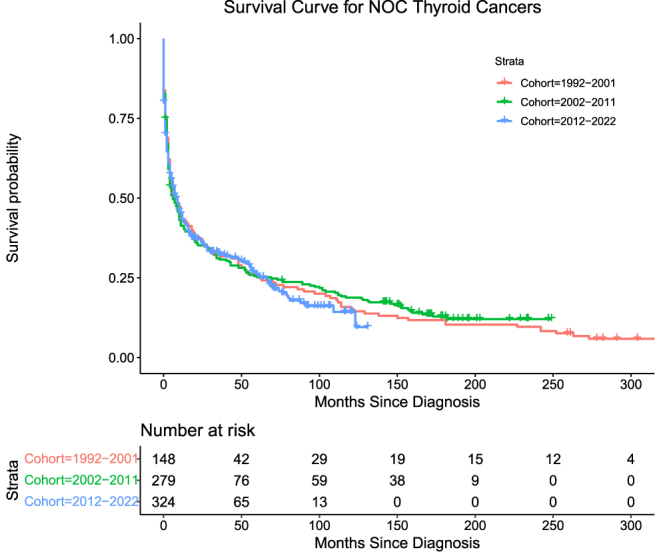
Survival curve for NOC thyroid cancers.

NOC thyroid cancers accounted for 38.3% (*n* = 751) of the cohort. Mortality for Groups 1, 2, and 3, respectively, is as follows: 107 (72.3%), 170 (60.9%), and 182 (56.2%) with log-rank (Mantel–Cox) = 1.399, *P* = 0.497. OTC exhibited moderate DSS across all three groups, with Groups 1–3 having similar rates of mortality (Table 5, Fig. 6).

## Discussion

In our study looking at DSS across three decades using the SEER database, we found that age, male sex, and year of diagnosis were significant predictors of mortality for all metastatic thyroid cancers. Among patients with differentiated tumors in our study, 71% of patients were over the age of 50, with patients in the 2012–2022 period being significantly older than patients from either the 1992 to 2001 or 2002 to 2011 time periods. Among patients with other cancers, patients in the 2012–2022 period were significantly older than those from either the 1992 to 2001 or 2002 to 2011 time periods, with 64.2% of patients in the 2012–2022 period being older than 65. In our subsequent survival analyses, increased HRs for older adults (namely, 65+) suggested older age being a significant predictor of mortality for both differentiated and other thyroid cancers. The observed increased mortality conferred by older age may be a result of earlier disease detection that reduces tumor burden in younger patients. Across all subtypes, female sex offered a minor but significant protective factor from mortality, in line with previous research on thyroid cancers (12).

Our findings demonstrate a modest but significant improvement in DSS for those diagnosed with metastasis of ATC, FTC (borderline significance), MTC, and OTC in the most recent decade (2012–2022) compared to earlier periods. Patients with MTC demonstrated an improvement in survival in the most recent time period, despite increased mortality associated with chemotherapy treatment (HR = 2.121), likely reflecting a confounding-by-indication effect (discussed further in the section titled ‘Limitations’). Patients with PTC and other thyroid cancers did not demonstrate any improvement in survival in the most recent decade cohort, although survival with PTC remained consistently high for all time periods.

We observed some changes in demographics over time. The majority of patients in the differentiated and other cohorts were female, although lower than the 3:1 female-to-male ratio commonly seen in thyroid cancers (13). Most patients were White and older. There were statistically significant differences observed across time periods with regard to age, sex, and race for differentiated cancers and significant differences observed for age and race for other thyroid cancers. Similarly, the proportion of non-White patients increased from the earliest to the latest time period, reflecting improved health equity and a more diverse patient population. Although we did not find an effect of race on survival in our study, it is well known that socioeconomic factors may impact access to care and health literacy, which may, in turn, influence mortality (14).

The incidence of thyroid cancers has tripled from the early 1980s to the mid-2010s (15). This increased incidence has been largely limited to wealthier nations (15, 16). Concerns over whether such drastic increases in thyroid cancer incidence are due to a truly increased incidence or simply overdiagnosis have prompted inquiry. A study in Switzerland found decreased mortality, increased incidence of early-stage or small thyroid tumors, and an increase in the number of thyroid resections, all of which could represent overdiagnosis (15). Early detection and treatment of thyroid tumors prevents further tumorigenesis and metastasis, potentially decreasing the number of metastatic cases and thereby increasing the overall survival of thyroid cancer patients.

A study from Wilhelm *et al.* in 2023 found the ten-year DSS to have remained constant in patients with metastatic thyroid carcinomas who were diagnosed between 1992 and 2008, with an overall mortality rate of 41.3% (17). The authors noted that the lack of adequate time between FDA approval of TKIs and that their last date of data collection may have influenced their findings (17). This group also found increases in differentiated thyroid carcinoma survival from the years 1992 to 2018, which they considered attributable to advances in diagnosis (17). Similarly, a study that assessed population-based thyroid cancer overall survival (OS) in a sample of 10,852 patients found a 6.29% increase in OS in patients diagnosed between 2017 and 2022 compared to 2012 and 2016 (18). Both of these studies mirror our findings of increased survival in more recent cohorts, which may be due to advances in early diagnosis. Our study’s long time period and use of SEER data collected up until 2022 may have allowed enough time since FDA approval to observe the therapeutic benefits from TKIs such as lenvatinib or sorafenib.

The advantage of targeted therapies such as TKIs is that they provide a means for interfering with the molecular mechanisms responsible for tumorigenesis (i.e. driving mutations in *BRAF*, *RAS*, or *RET)* that allow for uncontrolled cellular proliferation. PTC of 80% as well as large proportions of ATC are caused by mutations in proto-oncogenes such as *BRAF* and *RAS* (19, 20). *BRAF*-mutant thyroid cancers have been found to be more aggressive than *RAS*-mutant thyroid cancers due to decreased expression of genes necessary for iodine uptake, thereby enabling RAI resistance (21, 22). It follows then that therapies targeting such mutations provide a means by which to treat diseases that are resistant to traditional treatment modalities. Advances in treatment for both differentiated (e.g. lenvatinib and sorafenib) and other thyroid cancers (e.g. dabrafenib and trametinib) that target tyrosine kinase mutations have demonstrated promise in improving progression-free survival (PFS) in clinical trial settings (23).

It is possible that targeted therapies such as TKIs contributed to the increase in survival in distant disease in some of the thyroid cancer subtypes seen in this study, but without the granularity of data pertaining to patient treatment, intrinsic to SEER, this cannot be definitively stated. Early detection of thyroid cancer or a healthier patient population may have contributed to improvement in survival as well. Treatment data from SEER are non-specific, and associations with survival seen with radiation and chemotherapy may not be robust.

### Limitations

Despite our finding that some types of radiation therapy are associated with improved survival (HR < 1), radiation therapies in SEER encompass a wide range of modalities, and this finding may be more reflective of treatment selection or disease severity rather than any effect on survival. Also, patients who received radiation therapy may be fundamentally different than those who did not, with regard to tumor characteristics, comorbidities, or access to care.

Similarly, chemotherapy was also significantly associated with survival (HR < 1 for differentiated tumors, HR > 1 for other tumors, and HR = 2.121 for MTC). In SEER, the chemotherapy variable does not distinguish between cytotoxic chemotherapies and targeted therapies: a necessary distinction when interpreting its effect upon disease outcomes and survival. Instead, the lack of a protective response from the chemotherapy variable likely reflects ‘confounding-by-indication’, whereby patients who received some form of chemotherapy (e.g. cytotoxic, targeted, or immunotherapy) represent a subset of patients who likely suffered from highly aggressive disease variants that necessitated aggressive, systemic treatment. These patients would have possessed a substantially elevated mortality risk at baseline, and this elevated risk was, therefore, independent of any risk conferred by systemic treatment itself. This is further supported by an observable improvement in MTC survival at the population level that seemingly runs counter to the lack of protective effect from treatment. The identical coding of a plethora of treatment modalities prevents proper characterization of the impact of these variables on survival. We included these treatment modalities in our analysis for completeness, but the lack of granular treatment data in SEER must be acknowledged.

While our study provided insights into the evolving trends in thyroid cancer survival over the past thirty years in the United States, our study has limitations. First, the retrospective nature of the analysis and the reliance on SEER coding may have introduced bias related to data accuracy and completeness. Inclusion of data within the SEER database is dependent on accurate reporting and coding by various medical staff at various medical facilities, which may have inadvertently varied over time or by location. Second, the definitions of ‘distant’ cancer in the SEER database changed, most notably in 2004, becoming more restrictive. To gain as inclusive a sample as possible, three different definitions of ‘distant’ cancer were used, which may have introduced errors in both the consistency and the comparability of data across these different periods. Finally, as previously mentioned, the SEER database lacks detailed information on treatment, such as specific TKI, chemotherapy and radiation, patient comorbidities, and other factors that can affect the survival outcomes of patients.

## Conclusion

In summary, our study provides evidence supporting an improvement in survival outcomes in patients with metastatic thyroid cancer of the anaplastic, follicular, medullary, and oncocytic subtypes from 2012 to 2022. The findings may show only a coincidental increase in DSS in the time period associated with TKI use, but future studies using more granular datasets may provide more conclusive evidence for improvements in metastatic thyroid cancer survival stemming from TKI use. Continued advancements in targeted therapies and personalized medicine will serve to improve the prognosis and survival of patients with metastatic thyroid cancers.

## Declaration of interest

H Deshpande has a consulting or advisory status with honoraria for the following organizations: Aadi Bioscience, Blueprint Medicines, Daiichi Sankyo, Deciphera, Exelixis, and SpringWorks Therapeutics. All other authors have no conflicts of interest to report.

## Funding

This research did not receive any specific grant from any funding agency in the public, commercial, or not-for-profit sector.

## Ethical approval

The study does not require ethical approval due to the use of publicly available databases and deidentified patient data.
